# PD-L1 blockade exhibits anti-tumor effect on brain metastasis by activating CD8^+^ T cells in hematogenous metastasis model with lymphocyte infusion

**DOI:** 10.1007/s10585-021-10135-6

**Published:** 2021-11-19

**Authors:** Chinami Masuda, Mamiko Morinaga, Daiko Wakita, Keigo Yorozu, Mitsue Kurasawa, Masamichi Sugimoto, Osamu Kondoh

**Affiliations:** Product Research Department, Kamakura Research Laboratories, Chugai Pharmaceutical Co., Ltd, 200 Kajiwara, Kamakura, 247-8530 Japan

**Keywords:** Anti-PD-L1 antibody, CD8^+^ T cell, Non-small-cell lung cancer, Hematogenous brain metastasis model, Luciferase reporter

## Abstract

Brain metastases are common complication in cancer patients. Immune checkpoint inhibitors show therapeutic benefits also in patients with central nervous system (CNS) metastases. However, their antitumor effects on metastatic tumors and their underlying mechanisms are still poorly understood. In this study we investigated the antitumor effect of anti-programmed death-ligand 1 (PD-L1) antibody on metastatic brain tumors and evaluated immune responses during treatment. We employed a hematogenous brain metastasis xenograft model using immunodeficient mice with murine lymphocyte infusions. A human non-small-cell lung cancer (NSCLC) cell line stably expressing NanoLuc^®^ reporter (Nluc-H1915) was inoculated from the internal carotid artery of SCID mice. After metastases were established (24 days after inoculation), splenocytes prepared from H1915-immunized BALB/c mice were injected intravenously and mouse IgG or anti-PD-L1 antibody treatment was started (day 1). Evaluated by Nluc activity, tumor volume in the brain on day 14 was significantly lower in anti-PD-L1-treated mice than in mouse IgG-treated mice. Furthermore CD8^+^ cells were primarily infiltrated intratumorally and peritumorally and anti-PD-L1 treatment induced a significantly higher proportion of Granzyme B (GzmB)^+^ cells among CD8^+^ T cells. The antitumor effect of anti-PD-L1 antibody on brain metastasis is thought to be achieved by the enhanced activation of infiltrated CD8^+^ T cells into metastatic brain tumor. These results suggest that anti-PD-L1 antibody-containing regimens may be promising treatment options for cancer patients with brain metastases.

**Supplementary Information:**

The online version contains supplementary material available at 10.1007/s10585-021-10135-6.

## Introduction

Programmed death-ligand 1 (PD-L1) is an immune checkpoint molecule mainly expressed on tumor cells and immune cells that is involved in the suppression of cancer immunity. Anti-PD-L1 antibody relieves T cell suppression by inhibiting the binding of PD-L1 to programmed death-1 (PD-1) and B7.1 (also known as CD80), which are receptors on effector T cells, and thus exerts antitumor effects [1].

Brain metastases are thought to occur through seeding of circulating tumor cells into brain capillary vessels [2]. The rate of brain metastasis in end-stage non-small cell lung cancer (NSCLC) is reported to be up to 44% [3]. Brain metastasis is associated with extremely poor prognosis and quality of life [2, 4, 5]. The brain has a unique environment due to the blood–brain barrier (BBB) which limits the penetration of systemic therapeutic drugs [2]; the question of whether immune cells can cross BBB has remained controversial for decades [6].

Recently, however, immunotherapy has shown favorable data in the treatment of patients with brain metastases and has changed the conventional paradigm of cerebral immune privilege [6]. In a Phase III OAK study comparing atezolizumab (anti-PD-L1 antibody) with docetaxel (chemotherapeutic) in late-stage patients with previously treated NSCLC, subgroup analysis showed that, compared with docetaxel, atezolizumab also improved overall survival (OS) in asymptomatic patients with stable brain metastases who had previously received local treatment [7]. Subgroup analysis in the FIR study evaluating the efficacy and safety of atezolizumab in advanced NSCLC showed consistent results [8]. However, the antitumor effects of anti-PD-L1 antibody on metastatic brain tumors and the underlying immune system related mechanisms remain poorly identified.

To establish a brain metastasis model which can evaluate drug efficacy, we conducted preliminary studies with several cell lines. Nluc-H1915 is the only cell line that has been used to establish a hematogenous brain metastasis model that meets the requirements of selective tumor formation in the brain and an appropriate range in tumor size [9]. In the present study, we further extended this model to investigate whether cytotoxic T cells can access brain metastases in the brain parenchyma, and whether and how anti-PD-L1 antibody has antitumor effects on established brain metastases using a xenograft mouse model of hematogenous brain metastases with adoptive transfer of immune cells from tumor-immunized immuno-competent mice.

## Materials and methods

### Reagents

A monoclonal murine anti-PD-L1 monoclonal antibody (mAb; clone 6E11), which binds both human and mouse PD-L1, was provided by Genentech (South San Francisco, CA, USA). Mouse IgG was purchased from SouthernBiotech (Birmingham, AL, USA). Both were diluted with saline.

### Cell lines and culture conditions

The NCI-H1915 cell line, which is NSCLC cell line originally isolated from a brain metastasis [10], was obtained from American Type Culture Collection (ATCC; Manassas, VA, USA). According to the COSMIC database, Ras and p53 mutations were detected and EGFR mutations and ALK-EML4 translocations were not. It was maintained in RPMI-1640 (Sigma-Aldrich, St. Louis, MO, USA) supplemented with 10% (v/v) fetal bovine serum (Sigma-Aldrich or NICHIREI, Tokyo, Japan), 0.45% D-glucose (Sigma-Aldrich), 10 mM HEPES (Sigma-Aldrich), and 1 mM Na-pyruvate (Thermo Fisher scientific, Waltham, MA, USA).

The Nluc-H1915 cell line, which stably expresses the luciferase reporter gene secNluc, was prepared as described previously [9]. In brief, it was created by transfecting pEBMulti-secNluc plasmid into the human NSCLC cell line NCI-H1915 using FuGENE^®^ HD Transfection Reagent (Promega Corporation, Madison, WI, USA), and then stable cells were selected and maintained in the culture medium containing G418 Sulfate (Thermo Fisher Scientific).

The EMT6 cell line was purchased from ATCC and maintained in Waymouth’s Medium (Thermo Fisher Scientific) supplemented with 15% fetal bovine serum.

### Analysis of Nluc activity

Luminescence was measured using Nano-Glo^®^ Luciferase Assay System (Promega Corporation) according to the manufacturer’s instructions. A Varioskan Plate reader (Thermo Fisher Scientific Inc) was used to measure the luminescence.

### Laboratory animals

5–7-week-old male C.B-17/Icr-scid/scidJcl mice and 6–7-week-old male BALB/cA Jcl mice were obtained from CLEA Japan, Inc. (Tokyo, Japan). All animal experiments were reviewed and approved by the Institutional Animal Care and Use Committee at Chugai Pharmaceutical Co., Ltd., and conformed to the Guide for the Care and Use of Laboratory Animals published by the Institute of Laboratory Animal Resources.

### Preparation of a brain metastasis model and in vivo treatment

The brain metastasis model was prepared as described previously [9]. In brief, SCID mice were anesthetized by isoflurane and a microclamp was applied to the external carotid artery. Then Nluc-H1915 cells (1 × 10^5^ cells/head) were injected slowly into the internal carotid artery by surgical visualization, and finally the cut made in the skin was stitched up. Twenty-four days after tumor inoculation, blood was collected from the jugular vein and Nluc activity in plasma was measured and used to randomize mice into control and test groups (day 1). In parallel, spleens were harvested from BALB/c mice, which were subcutaneously immunized with H1915 cells one week before, and splenocytes were prepared with Hank’s balanced salt solution (Sigma-Aldrich) after treatment with VersaLyse lysing solution (Beckman Coulter, Brea, CA, USA). This splenocytes (5 × 10^4^ cells /head) were intravenously injected into the tail of brain metastasis model mice as donor lymphocyte infusion (DLI) on day 1 and mouse IgG and anti-PD-L1 antibody were administered intraperitoneally at a dose of 10 mg/kg twice a week.

### Protein extraction

Brains were removed from mice after euthanasia, frozen immediately in liquid nitrogen, and stored at − 80 °C until use. The brain parenchyma cells were homogenized in cell lysis buffer (Cell Signaling Technology, Danvers, MA, USA) and the supernatant in each homogenate was collected after centrifugation and used for analyses.

### Flowcytometry analysis

Brains were excised after euthanasia and single-cell suspensions were obtained by mincing brain and digestion with a gentleMACS Dissociator and a Tumor Dissociation Kit, human (Miltenyi Biotec, Bergisch Gladbach, Germany). Single-cell suspensions were incubated with anti-Fcγ receptor antibody (Clone 2.4G2, Tonbo Biosciences, San Diego, CA, USA) and the fixable viability dye FVD780 (Thermo Fisher Scientific), then stained with the following monoclonal antibodies; mouse CD45 (30-F11), CD3 (17A2), CD49b (DX5), CD8 (KT15), CD4 (GK1.5), Foxp3 (FJK-16s), granzyme B (GB11), CD69 (H1.2F3), Ki67 (B56), PD-1(29F.1A12), human EGFR (AY13), human PD-L1 (MIH1 or 29E.2A3) from BD Biosciences (San Jose, CA, USA), BioLegend (San Diego, CA, USA), Thermo Fisher Scientific, or Medical & Biological Laboratories (Nagoya, Japan). The appropriate conjugated isotype-matched IgG was used as the control if necessary. Intracellular staining was performed using a Foxp3/Transcription Factor Staining Buffer Set (Thermo Fisher Scientific). Stained cells were analyzed using LSRFortessa X-20 cell analyzer (BD Biosciences) and data analyzed with FlowJo 10 software (Tree Star, San Carlos, CA, USA). The gating strategies were shown in Supplementary Figs. S1 and S2.

### Immunohistochemistry analysis

Brains were removed and sliced into six sections. Nluc-H1915 cells in the brain were stained by IHC with an anti-human EGFR antibody, EGF Receptor (D38B1) XP^®^ Rabbit mAb (Cell Signaling Technology).

Localization of CD8^+^ cells in brain were evaluated by immunohistochemical staining of CD8α using CD8α (D4W2Z) XP^®^ Rabbit mAb (Mouse Specific) (Cell Signaling Technology).

### In vitro co-culture experiments

Nuc-H1915 cells and EMT6 cells were pretreated with human and mouse IFN-γ, respectively (Peprotech, Cranbury, NJ, USA). Spleens were harvested from BALB/c mice immunized with H1915 cells subcutaneously a week before. After treatment with VersaLyse lysing solution, splenocytes were co-cultured with Nluc-H1915 cells or EMT6 cells treated with Mytomycin C (Nacalai Tesque, Kyoto, Japan) at the E/T ratio = 10 in 96-well U-bottom plate for 2 days. Cells were analyzed using flowcytometry as described above and IFN-γ in the supernatant were determined by using Mouse IFN-γ Quantikine ELISA Kit (R&D Systems, Minneapolis, MN, USA) according to the manufacturer’s protocol.

### Statistical analysis

Statistical differences between groups were assessed using Wilcoxon’s signed-rank test, Wilcoxon’s rank sum test, or Tukey’s HSD test. All reported p values were two-sided, and p < 0.05 was considered to be statistically significant. All statistical analyses were conducted using the JMP^®^ Version 15 software (SAS Institute Inc., Cary, NC, USA).

## Results

### The effects of anti-PD-L1 antibody on the brain metastasis

First, we examined the antitumor effects of DLI with or without anti-PD-L1 antibody administration on established brain metastases.

**Fig. 1:**
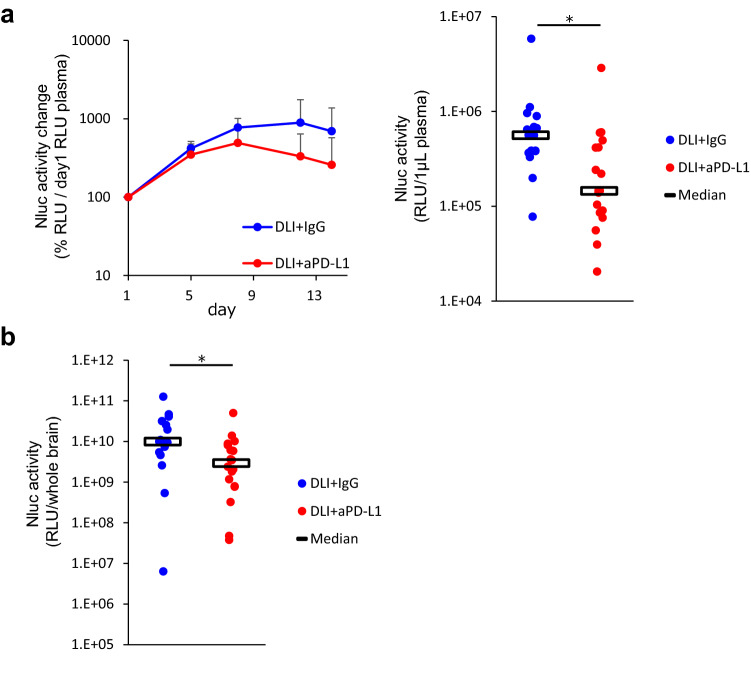
Antitumor effects of anti-PD-L1 antibody on established brain metastasis. Donor lymphocyte infusion were performed on day 1, and anti-PD-L1 antibody or mouse IgG (10 mg/kg) was administered intraperitoneally into brain metastasis model mice twice a week (n = 16 or 18/group). Three independent experiments were pooled and analyzed. **a** Tumor volume change was evaluated by measuring Nluc activity in plasma. In the left panel, plasma Nluc activity relative to day 1 was calculated. Data represent mean + SD. In the right panel, Antitumor activity of anti-PD-L1 antibody in whole body was evaluated by measuring the plasma Nluc activity on day 14 (relative light unit/1 μL plasma). **b** Antitumor activity of anti-PD-L1 antibody on brain metastasis was evaluated by measuring Nluc activity (relative light unit/whole brain) in the supernatant of brain homogenates on day 14. Dots indicate individuals and bars represent median. Statistical differences are shown as *p < 0.05 (assessed by Wilcoxon’s rank sum test)

To evaluate tumor volume changes indirectly, we monitored plasma Nluc activity, which reflects the amount of tumor in the whole body. Nluc activity in plasma was observed to first increase and then to decrease during treatment in both the DLI + IgG and DLI + anti-PD-L1 groups. In DLI + IgG group, it increased from day 1 to 8 and decreased from day 12 to 14. In DLI + anti-PD-L1 group, it increased from day 1 to 8 and decreased from day 8 to 14, as assessed by Wilcoxon signed rank test (Fig. 1a, left panel). On day 14, plasma Nluc activity was significantly lower in the DLI + anti-PD-L1 than in DLI + IgG group (Fig. 1a, right panel). The antitumor effect of anti-PD-L1 antibody on brain metastasis was determined by comparing Nluc activity in the brains of DLI + anti-PD-L1 antibody- and DLI + mouse IgG-treated mice on day 14. The Nluc activity in the DLI + anti-PD-L1 antibody-treated group was significantly reduced (Fig. 1b), which indicates that anti-PD-L1 antibody has an anti-tumor effect on the brain metastases in this model.

### The antitumor effect of anti-PD-L1 antibody on the brain metastasis model was DLI dependent

Nluc-H1915 cells expressed PD-L1 on the cell surface in vitro and in vivo. Flow cytometry analysis (Supplementary Fig. S3) showed that the overall PD-L1 expression levels on tumor cells were not remarkably different among all animals tested; the PD-L1 positivity rate (compared to isotype control) of cancer cells in brain in all experimental animals and their gMFI (geometric Mean Fluorescence Intensity) were at similar levels. To confirm that the antitumor effect was not caused by anti-PD-L1 antibody alone but was dependent on DLI, we administered IgG or anti-PD-L1 antibody to brain metastasis model mice without DLI.

**Fig. 2:**
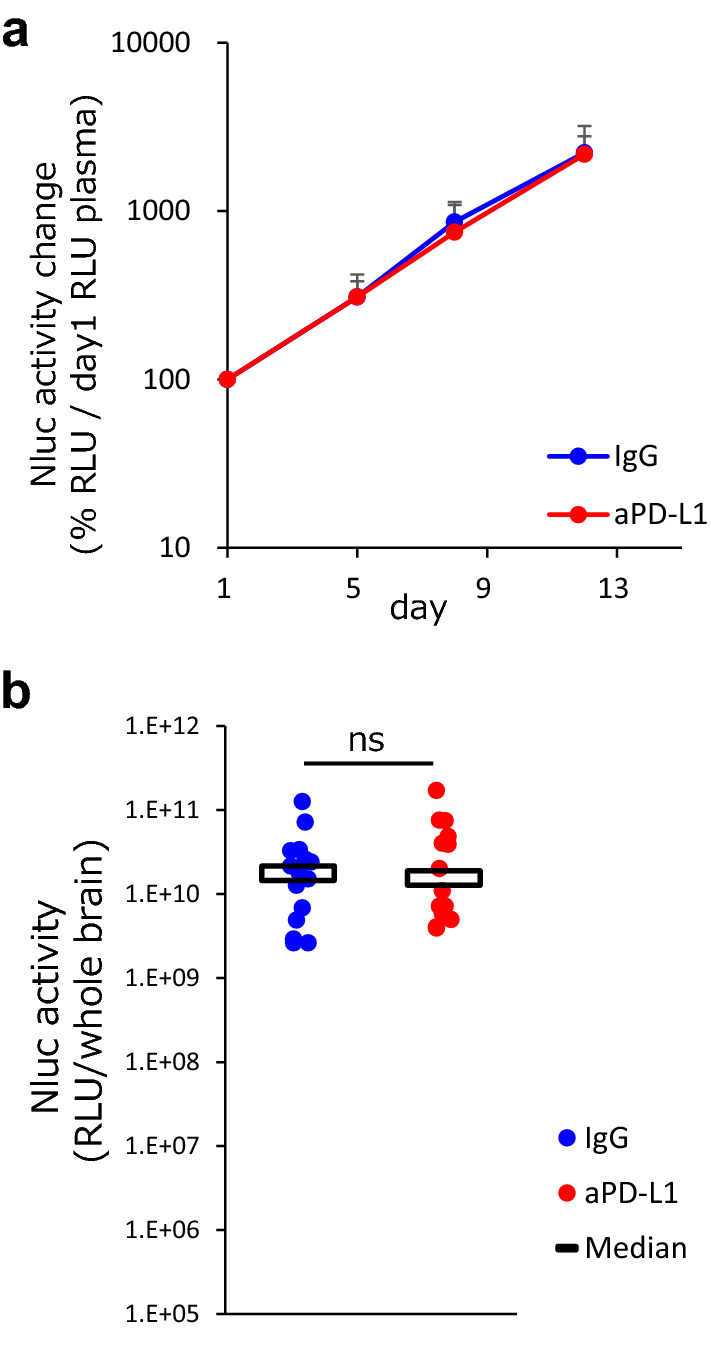
The antitumor effect of anti-PD-L1 antibody on the brain metastasis model was donor lymphocyte infusion dependent. Anti-PD-L1 antibody or mouse IgG (10 mg/kg) was administered intraperitoneally into brain metastasis model mice twice a week without donor lymphocyte infusion (n = 14 or 15/group). Two independent experiments were pooled and analyzed. **a** Tumor volume change was evaluated by measuring Nluc activity in plasma and plasma Nluc activity relative to day 1 was calculated. Data represent mean + SD. **b** Antitumor activity of anti-PD-L1 antibody on brain metastasis was evaluated by measuring Nluc activity (relative light unit /whole brain) in the supernatant of brain homogenates on day 12. Dots indicate individuals and bars represent median. Statistical differences are determined as p < 0.05, *ns* not significant (assessed by Wilcoxon’s rank sum test)

Plasma Nluc activity monotonically increased in both groups (between continuous timepoints, assessed by Wilcoxon signed rank test) (Fig. 2a) and was consistent with plasma Nluc activity. There was no significant difference in brain Nluc activity on day 12 between the two groups (Fig. 2b).

These results indicate that the antitumor effect of anti-PD-L1 antibody on the brain metastasis in this model was DLI dependent.

### Treatment with anti-PD-L1 antibody enhanced activation of CD8^+^ T cells in the metastatic brain tumor

To investigate the mechanism of anti-PD-L1 antibody in antitumor effects with DLI, we evaluated infiltration of immune cells in the brain using flowcytometry on day 11 and IHC on day14.

**Fig. 3:**
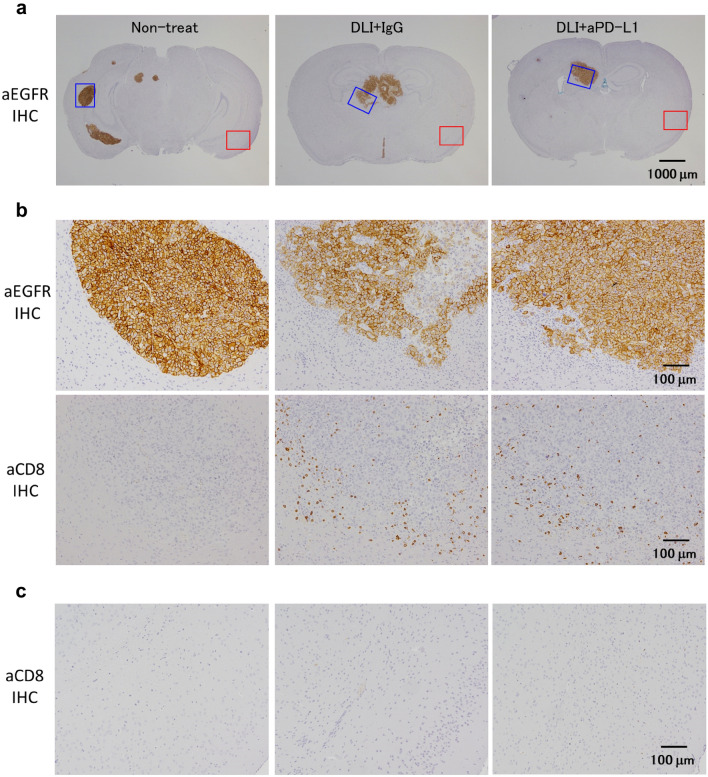
CD8^+^ cells localized intertumoral and contiguous peritumoral stroma of the metastatic brain tumor. donor lymphocyte infusion + anti-PD-L1 antibody or mouse IgG (10 mg/kg) was administered intraperitoneally into brain metastasis model mice twice a week. Brains were removed from the mice on day 14. Brain slices were stained by immunohistochemistry using anti-human EGFR antibody (for tumor cells) and anti-mouse CD8 antibody. **a** Representative micrographs at low magnification. **b** High magnification micrographs of tumor sites indicated in (**a**) as blue squares. **c** High magnification micrographs of non-lesion sites indicated in (**a**) as red squares. Scale bars are 1000 μm (top panel) and 100 μm (middle and lower panel), respectively

**Fig. 4:**
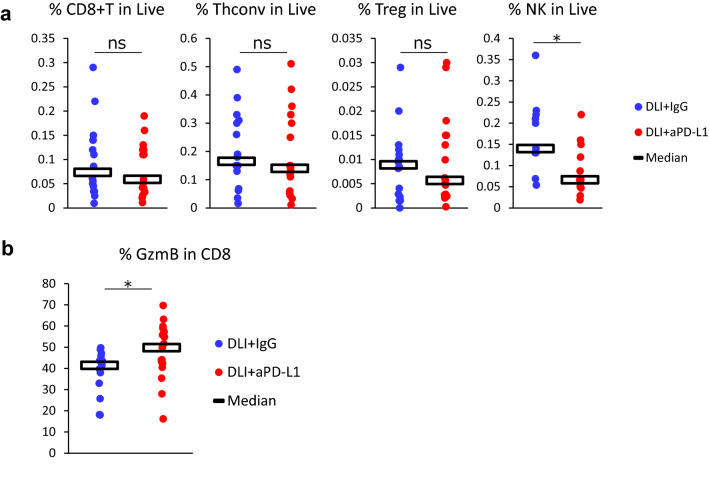
Treatment with anti-PD-L1 antibody enhanced activation of CD8^+^ T cells in the metastatic brain. Donor lymphocyte infusion + anti-PD-L1 antibody or mouse IgG (10 mg/kg) was administered intraperitoneally into brain metastasis model mice twice a week. Brains were removed from the mice on day 11 (n = 16 or 17/group; for NK cells, n = 11 or 12/group). Two or three independent experiments were pooled and analyzed. **a** Proportion of CD8^+^ T cells, conventional helper T cells, regulatory T cells, and Natural killer cells in live cells in brain were analyzed using flowcytometry. Dots indicate individuals and bars represent median. **b** Proportion of Granzyme B^+^ cells in CD8^+^ T cells. Statistical differences are shown as *p < 0.05, *ns* not significant (assessed by Wilcoxon’s rank sum test)

As previously reported [9], tumor regions in the brain consisting of Nluc-H1915 cells were identified as EGFR^+^ areas by IHC staining (Fig. 3a). These were consistent with pathological areas identified by HE staining (data not shown). IHC staining that CD8^+^ cells were primarily infiltrated intratumoral and contiguous peritumoral stroma (Fig. 3b). No CD8^+^ cells were observed in non-lesion sites away from the brain metastases (Fig. 3c). Quantitatively evaluated by flowcytometry, anti-PD-L1 had no effect of the proportion of CD8^+^ T cells (Fig. 4a). We also assessed other immune cell subsets and found no significant difference in conventional helper T (Thconv) cells, and regulatory T (Treg) cells to live cells in the whole brain, while natural killer (NK) cells were significantly decreased in the DLI + aPD-L1 group (Fig. 4a). The proportion of GzmB^+^ cells in CD8^+^ T cells was significantly higher in the anti-PD-L1 group than in the mouse IgG group (Fig. 4b).

These results suggest that the antitumor effect of the anti-PD-L1 treatment was achieved by enhanced activation of CD8^+^ T cells in metastatic brain tumor.

### Anti-PD-L1 antibody treatment enhanced activation of CD8^+^ T cells in response to H1915 in vitro

To investigate whether this activation of CD8 cells occurred in response to H1915 cells, splenocytes prepared in the same way as DLI were co-cultured with Nluc-H1915 (target) cells. To confirm that the response was not non-specific, EMT6 cells with matched MHC with DLI were set as a negative control. EMT6 expresses PD-L1 after stimulation with IFN-γ (data not shown). They were co-cultured in the presence of anti-PD-L1 antibody for 2 days and T cell activation was analyzed using flowcytometry and ELISA.

**Fig. 5:**
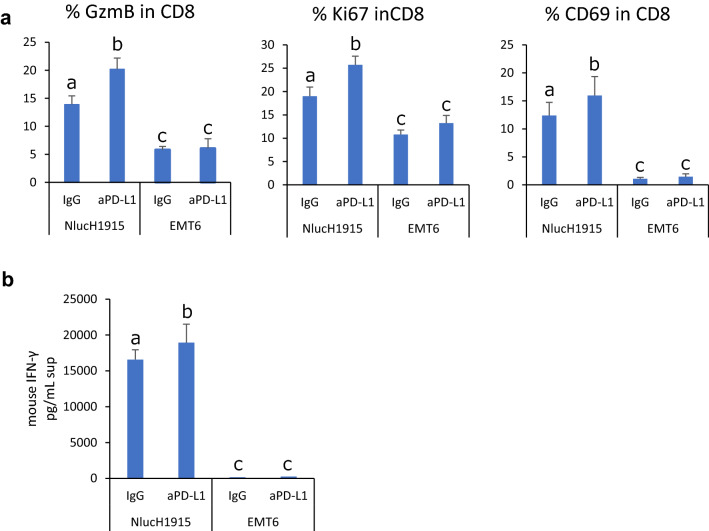
Anti-PD-L1 antibody treatment enhanced activation of CD8^+^ T cells in response to H1915 in vitro. Splenocytes prepared in the same way as donor lymphocyte infusion and target NlucH1915 cells or non-target EMT6 cells were co-cultured for 2 days in the presence of anti PD-L1 antibody. **a** Activation markers of CD8^+^ T cells were assessed using flowcytometry. **b** IFN-γ production in culture supernatant was measured by ELISA. Data represent mean + SD of 6 wells/condition. Different letters indicate statistical significance. p < 0.05 (assessed by Tukey’s HSD test)

In the presence of control IgG, the proportion of GzmB^+^, CD69^+^, Ki67^+^ in CD8^+^ T cells were significantly higher when co-cultured with NlucH1915 than when co-cultured with EMT6. PD-L1 blockade significantly enhanced the activation of CD8^+^ T cells co-cultured with Nluc-H1915, but not with EMT6. (Fig. 5a) Similar results were obtained with IFN-γ levels in supernatant (Fig. 5b).

These results indicate that CD8^+^ T cells in splenocytes from H1915-immunized mice were re-activated in response to Nluc-H1915 cells and that PD-L1 blockade promoted the activation, suggesting that anti-PD-L1 treatment in vivo enhanced the immune response of transferred CD8^+^ T cells against Nluc-H1915.

## Discussion

In this study, we demonstrated the antitumor effect of anti-PD-L1 antibody with lymphocyte infusion on established brain metastases in a hematogenous brain metastasis xenograft model using the human NSCLC cell line, Nluc-H1915. Without DLI, no antitumor effect of anti-PD-L1 was observed, suggesting that the effect of anti-PD-L1 is not due to the direct induction of cell death on cancer cells or as a result of mechanisms solely mediated by NK cells and macrophages in the host SCID mice. CD8+ cells localized in the intertumoral and contiguous peritumoral stroma where they were activated upon contact with H1915 cells. These tumor-recognizing CD8+ cells may exert an antitumor effect that is significantly enhanced by anti-PD-L1 antibody. It has been reported that NK cells, in addition to CD8+ T cells, mediated the antitumor effect of PD-1/PD-L1 blockade immunotherapy [11]. In this study, although the percentage of NK cells was decreased in anti PD-L1 treated group, enhanced antitumor effect still exerted, suggesting that NK cells are not the main effector of the antitumor effect.

There are several limitations in this experimental model. First, this model employed human cancer cells, and the responses by immune cells transferred from mice represent a xenoreaction to a heterologous antigen. Second, in this study, immune cells are activated in the donor and then transferred to the recipient with brain metastasis, and our research mainly focused on the effector phase of the cancer-immunity cycle [12]. These points need to be further investigated using an immunocompetent brain metastasis model.

While the CNS has long been thought to have immune privilege, recent studies reported the discovery of functional lymphatic vessels and antigen drainage [13], immune cell trafficking from the brain [14] and immune surveillance of the CNS in meninges [15]. These findings have been making it more clear how the antigens in CNS are presented. On the other hand, the cytotoxic response, a possible mechanism of action of immune checkpoint inhibitors, requires CD8^+^ T cells to migrate across blood vessels and come into contact with target cells [16]. It has been reported that BBB regulates the transport of various molecules [17], which may block cell trafficking as well as antibody drug penetration. It is possible that the formation of metastatic tumors alters the properties of the BBB, resulting in a condition known as blood–brain tumor barrier (BTB). In BTB, the BBB is partially disrupted and more permeable than in the normal brain [18], yet it is not sufficiently or homogeneously permeable for effective drug therapy [19, 20]. In this study, we used Nluc-H1915 cells because they are expected to reflect the microenvironment of the brain metastases of human patients due to their clinical origin. Furthermore, brain metastatic nodules were formed by injecting tumor cells via the internal carotid artery. This process recapitulates major steps in the establishment of hematogenous brain metastases [21], which theoretically suggests that the property of the BBB/BTB in this model imitates well that in patients with brain metastasis. When assessed by IHC, CD8^+^ cells of DLI localized intratumoral and contiguous peritumoral stroma suggest that activated CD8^+^ T cells can infiltrate beyond the BBB/BTB into metastases located in the brain parenchyma. The exact condition of BBB/BTB in metastatic brain nodules and the detailed mechanism of recruitment and infiltration of CD8^+^ cells across the BBB/BTB (e.g. tethering, adhesion to the brain vasculature, transmigration) need further investigation.

In addition to anti-PD-L1, anti-PD-1 is also widely used in clinical practice, and it will be interesting to see if anti-PD-1 has the same biological effect as anti-PD-L1 in this study. Both of them inhibit PD-1**-**PD-L1 interaction, but PD-L1 blockade additionally inhibits *cis* interaction between PD-L1 and CD80 [22], and PD-1 blockade inhibits interaction between PD-L2 and PD-1 [23]. It has been reported that human PD-L1 and mouse PD-1 have cross-reactivity [24], and it is therefore highly probable that human PD-L1 expressed by Nluc-H1915 and mouse PD-1 expressed by infused lymphocytes interacted in this model. On the other hand, it has not been reported about interaction between human PD-L2 and mouse PD-1. Therefore, it is difficult to properly evaluate the potential difference between the two in the current study. This needs to be further investigated using a syngeneic brain metastasis model.

In clinical practice, PD-L1 expression in tumors is assessed to determine therapeutic strategies, but biopsies from brain metastases are rarely used for this diagnosis. Also, the PD-L1 status of brain metastases and primary lesions is not always the same [25]. Therefore, it is unclear whether a diagnosis based on PD-L1 expression in the primary lesion can predict the response in brain metastatic lesions, but investigating the correlation/difference in PD-L1 expression between brain metastases and primary leads to predicting the clinical benefit of brain metastases. And we think it is a future issue, including clinical practice.

In conclusion, the outcomes of this study suggest that anti-PD-L1 antibody has anti-tumor effect on metastatic brain tumor, and that regimens containing anti-PD-L1 antibody may be promising therapeutic options for patients with brain metastases.

## Supplementary Information

Below is the link to the electronic supplementary material.

Supplementary file1 (PDF 951 KB)

## Data Availability

The datasets generated during and/or analyzed during the current study are available from the corresponding author on reasonable request.

## Abbreviations

BBB: Blood–brain barrier
BTB: Blood–brain tumor barrier
CNS: Central nervous system
DLI: Donor lymphocyte infusion
HE: Hematoxylin and eosin
IgG: Immunoglobulin G
IHC: Immunohistochemistry
mAb: Monoclonal antibody
NK: Natural killer
NSCLC: Non-small-cell lung cancer
PD-1: Programmed death-1
PD-L1: Programmed death-ligand 1
RLU: Relative light unit
Thconv: Conventional helper T
Treg: Regulatory T
