# Supplementary material for: PD-L1 blockade exhibits anti-tumor effect on brain metastasis by activating CD8+ T cells in hematogenous metastasis model with lymphocyte infusion
Source: Clin Exp Metastasis. 2021 Nov 19;39(2):335–44. doi: 10.1007/s10585-021-10135-6 (PMC8971192; doi:10.1007/s10585-021-10135-6)
Supplement: Supplementary file 1 — Supplementary file1 (PDF 951 KB) [file 10585_2021_10135_MOESM1_ESM.pdf]

PD-L1 blockade exhibits anti-tumor effect on brain metastasis by activating CD8$^+$ T cells in hematogenous metastasis model with lymphocyte infusion

Supplementary information

Chinami Masuda[†], Mamiko Morinaga[†], Daiko Wakita, Keigo Yorozu, Mitsue Kurasawa, Masamichi Sugimoto, Osamu Kondoh

[†]: these authors equally contributed

Product Research Department, Kamakura Research Laboratories, Chugai Pharmaceutical Co., Ltd.

200 Kajiwara, Kamakura, 247-8530, Japan

Corresponding author:

Masamichi Sugimoto

Email: sugimotomsm@chugai-pharm.co.jp

Supplementary Figure 1

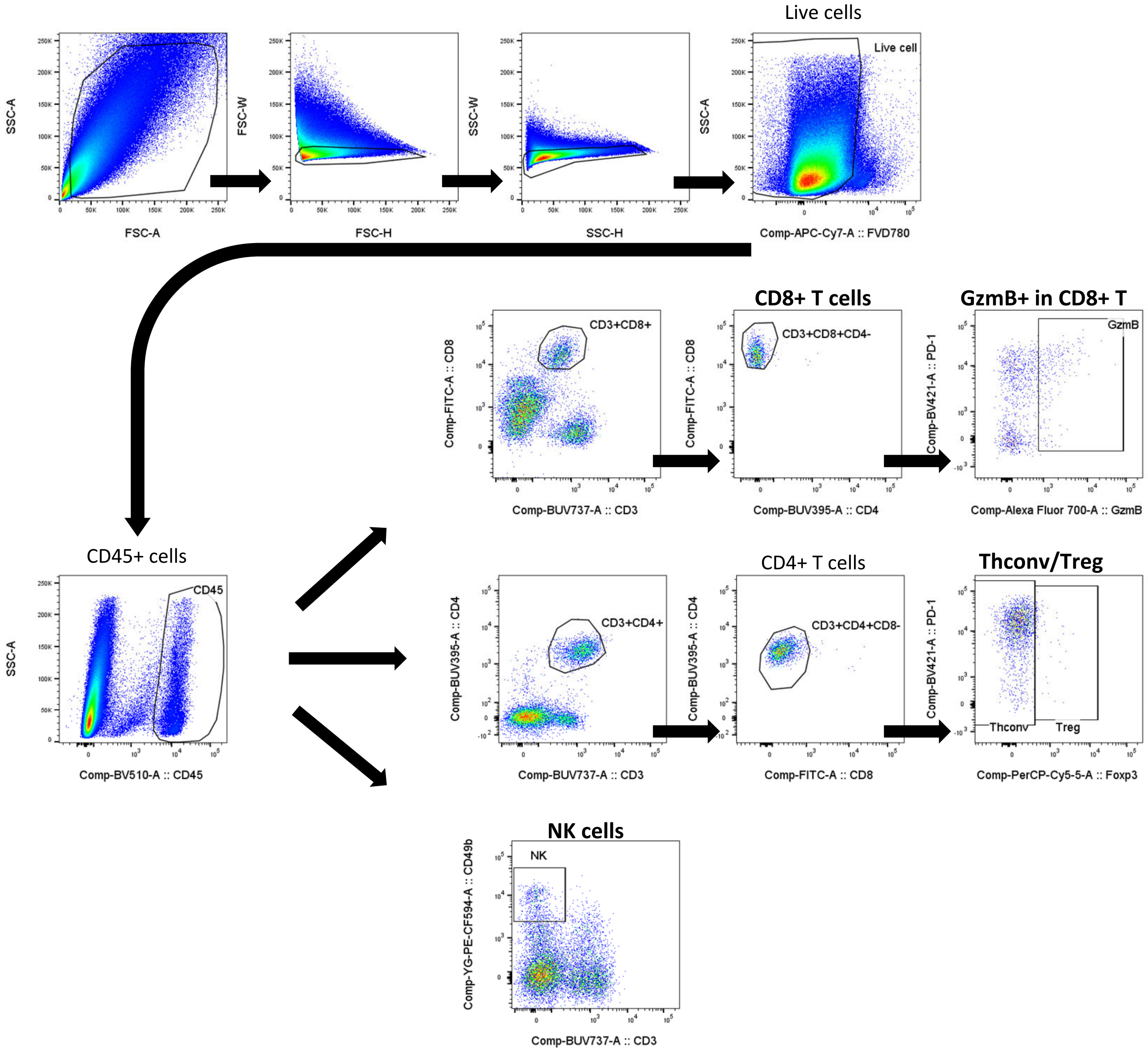

Supplementary Figure 1 Gating strategy for flow cytometric analysis of T cell subsets and NK cells in brain

Cells were gated based on forward (FSC) and side scatter (SSC) profiles followed by exclusion of doublets. Dead cells were excluded with Fixable Viability Dye (FVD) and CD45 was used to identify leucocytes. CD8+ T cells (CD3+ CD8+ CD4-), Thconv cells (CD3+ CD4+ CD8- Foxp3-), Treg cells (CD3+ CD4+ CD8- Foxp3+) and NK cells (CD3- CD49b+) were gated. Representative plots with gates were shown.

Supplementary Figure 2

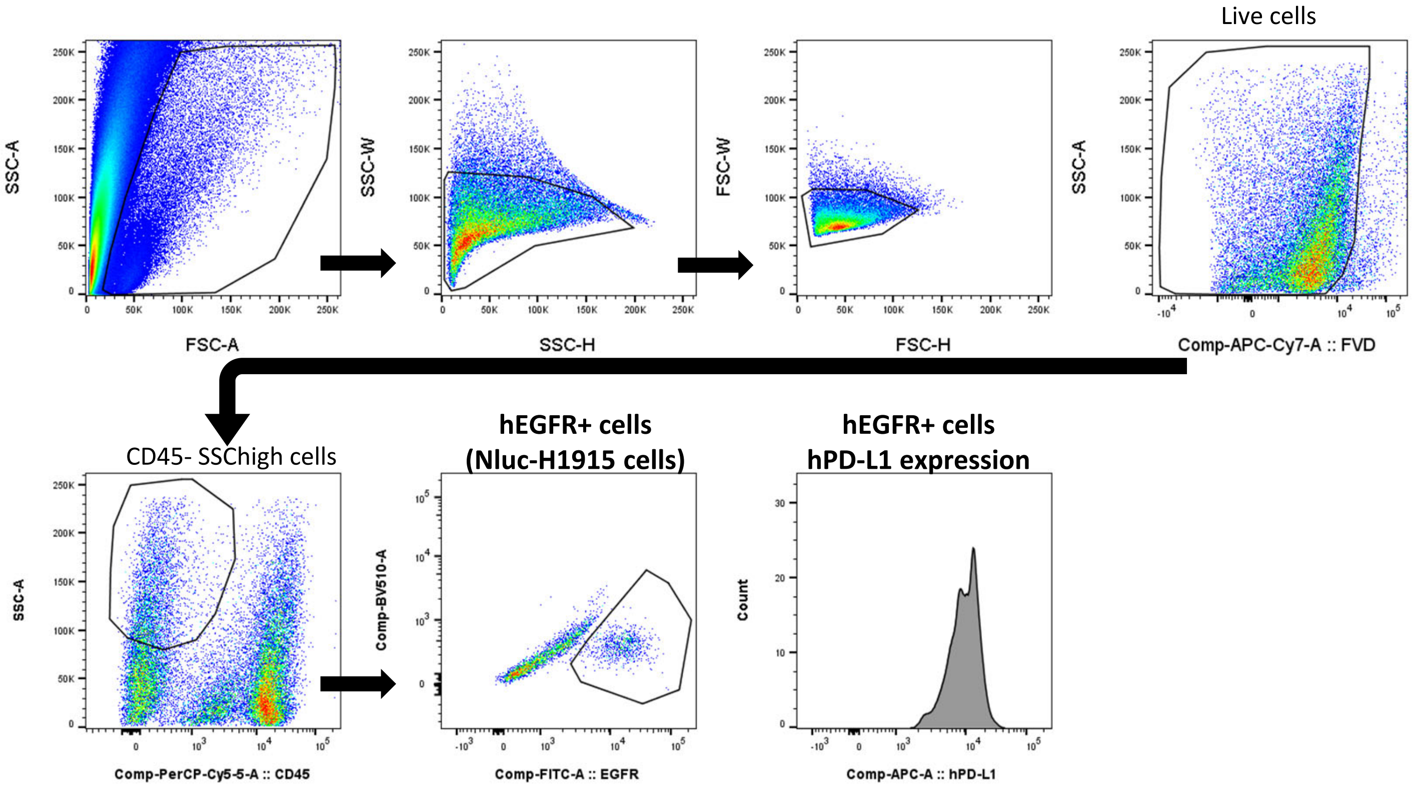

Supplementary Figure 2 Gating strategy for flow cytometric analysis of Tumor cells in brain

Cells were gated based on forward (FSC) and side scatter (SSC) profiles followed by exclusion of doublets. Dead cells were excluded with Fixable Viability Dye (FVD). Nluc-H1915 cells (CD45-, SSC-high, hEGFR+) were gated to evaluate PD-L1 expression. Representative plots with gates were shown.

Supplementary Figure 3

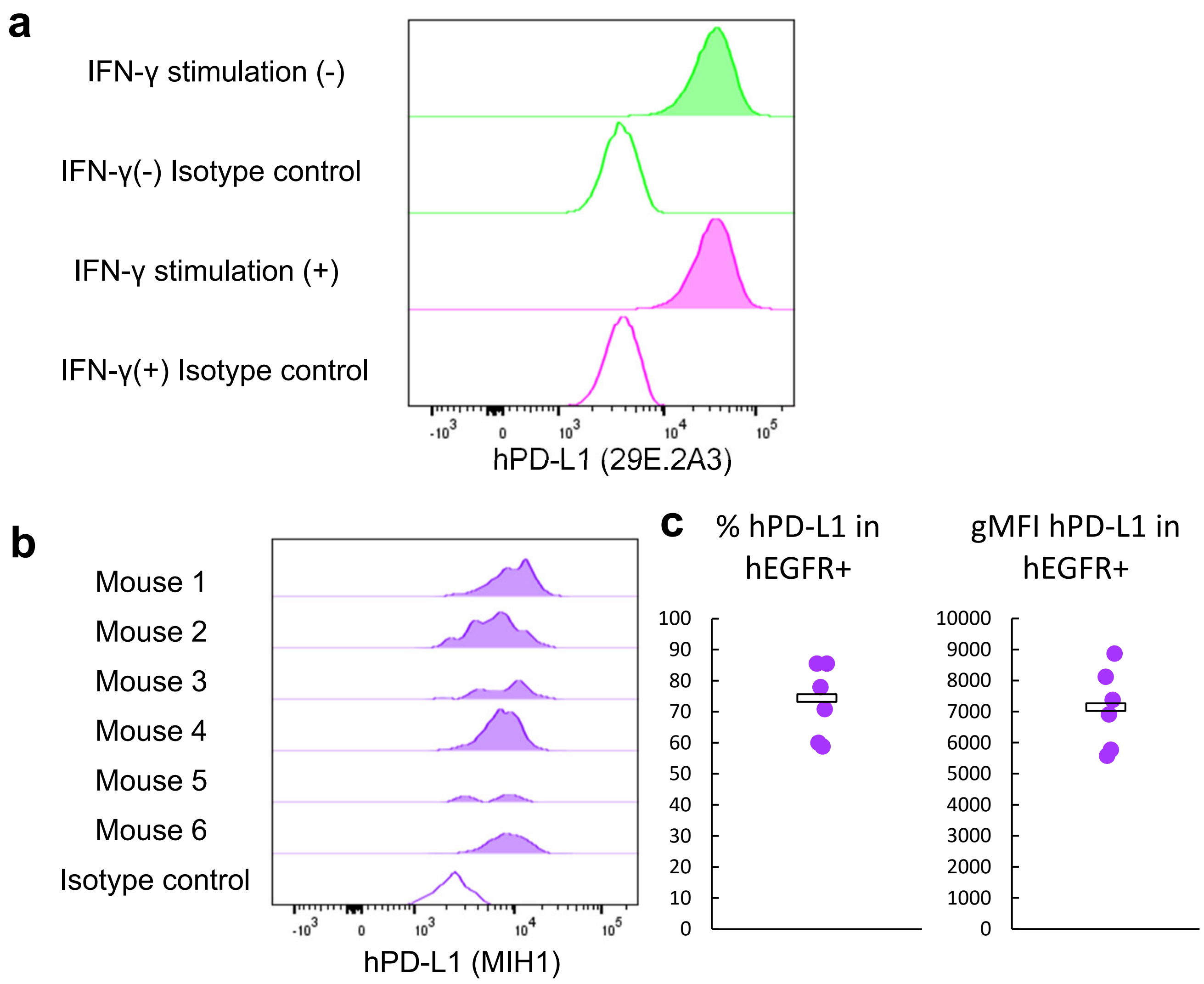

Supplementary Figure 3 Nluc-H1915 cells expressed PD-L1 on the cell surface in vitro and in vivo brain metastatic tumor

a: Nluc-H1915 cells were cultured in vitro and human PD-L1 expression on the cell surface was assessed using flowcytometry. IFN-γ stimulation (+); Nluc-H1915 cells were stimulated with 20 ng/mL human IFN-γ on the previous day of analysis.

b: donor lymphocyte infusion was performed on day 1, and mouse IgG was administered intraperitoneally into brain metastasis model mice twice a week. Brains were removed from the mice on day 11 and analyzed using flowcytometry. Nluc-H1915 cells were identified as human $EGFR^+$, and human PD-L1 expression was assessed. Each histogram indicates an individual and blank indicates isotype control.

c: the PD-L1 positivity rate (compared to isotype control) of human $EGFR^+$ cells in brain and their gMFI (geometric Mean Fluorescence Intensity). Dots indicate individuals and bar indicates median.
